# Reliable Metabolic Flux Estimation in *Escherichia coli* Central Carbon Metabolism Using Intracellular Free Amino Acids

**DOI:** 10.3390/metabo4020408

**Published:** 2014-05-30

**Authors:** Nobuyuki Okahashi, Shuichi Kajihata, Chikara Furusawa, Hiroshi Shimizu

**Affiliations:** 1 Department of Bioinformatic Engineering, Graduate School of Information Science and Technology, Osaka University, 1-5 Yamadaoka, Suita, Osaka 565-0871, Japan; E-Mails: n-okahashi@ist.osaka-u.ac.jp (N.O.); s-kajihata@ist.osaka-u.ac.jp (S.K.); chikara.furusawa@riken.jp (C.F.); 2 Quantitative Biology Center, RIKEN, 6-2-3 Furuedai, Suita 565-0874, Japan

**Keywords:** metabolic flux analysis, intracellular free amino acids, *Escherichia coli*, GC-MS

## Abstract

^13^C metabolic flux analysis (MFA) is a tool of metabolic engineering for investigation of *in vivo* flux distribution. A direct ^13^C enrichment analysis of intracellular free amino acids (FAAs) is expected to reduce time for labeling experiments of the MFA. Measurable FAAs should, however, vary among the MFA experiments since the pool sizes of intracellular free metabolites depend on cellular metabolic conditions. In this study, minimal ^13^C enrichment data of FAAs was investigated to perform the FAAs-based MFA. An examination of a continuous culture of *Escherichia coli* using ^13^C-labeled glucose showed that the time required to reach an isotopically steady state for FAAs is rather faster than that for conventional method using proteinogenic amino acids (PAAs). Considering 95% confidence intervals, it was found that the metabolic flux distribution estimated using FAAs has a similar reliability to that of the PAAs-based method. The comparative analysis identified glutamate, aspartate, alanine and phenylalanine as the common amino acids observed in *E. coli* under different culture conditions. The results of MFA also demonstrated that the ^13^C enrichment data of the four amino acids is required for a reliable analysis of the flux distribution.

## 1. Introduction

Metabolic flux analysis (MFA) is a tool driving metabolic engineering through a detailed understanding of intracellular carbon flux distributions in various organisms [1,2,3,4,5]. The level of flux in each reaction is estimated by a tracer labeling experiment using, such as ^13^C-labeled glucose, as the labeling patterns of the intracellular metabolites reflect the flux distribution inside the cells [6,7,8,9]. In conventional MFA, the flux distribution is estimated from the ^13^C enrichment of proteinogenic amino acids (PAAs) determined by gas chromatography-mass spectrometry (GC-MS) or nuclear magnetic resonance (NMR) [10,11]. Whereas a large amount of PAAs in the cells is preferable for a precise determination of the ^13^C enrichment, the slow turnover of PAAs has restricted MFA applications, since a relatively long experimental time, for example, 5 residence time in continuous culture, is required for the complete labeling of the PAAs.

For the MFA study of batch and fed-batch culture, intracellular free amino acids (FAAs) with faster turnover rates are more promising targets for a ^13^C enrichment measurement [12,13,14,15,16]. The time course analysis of ^13^C enrichment of FAAs demonstrated that the experimental time required to reach an isotopic steady state of the FAAs is 2 to 10 times faster than that for PAAs [17]. However, the smaller pool sizes and large compositional variations of FAAs are expected to affect the design of an MFA experiment [18]. This is because a confidence interval of flux estimation depends on a set of measureable FAAs. The relationship between the set of measurable data and resulting confidence intervals are not well investigated from the aspect of experimental data. The large sampling volume of broth required for broader amino acid coverage would disturb a constant culture condition. Understanding the required dataset for reliable MFA will be a useful guideline for the design of MFA experiments using FAAs.

In this study, minimal ^13^C enrichment data of FAAs to perform the FAAs-based MFA was investigated by the analysis of *Escherichia coli*. It was realized that four amino acids, i.e., glutamate (Glu), aspartate (Asp), alanine (Ala), and phenylalanine (Phe), are the most commonly observed in *E. coli* at distinct culture conditions. The results of MFA also demonstrated that the ^13^C enrichment data of the four amino acids is required for a reliable analysis of the flux distribution.

## 2. Results and Discussion

### 2.1. Continuous Culture of E. coli MG1655

**Figure 1:**
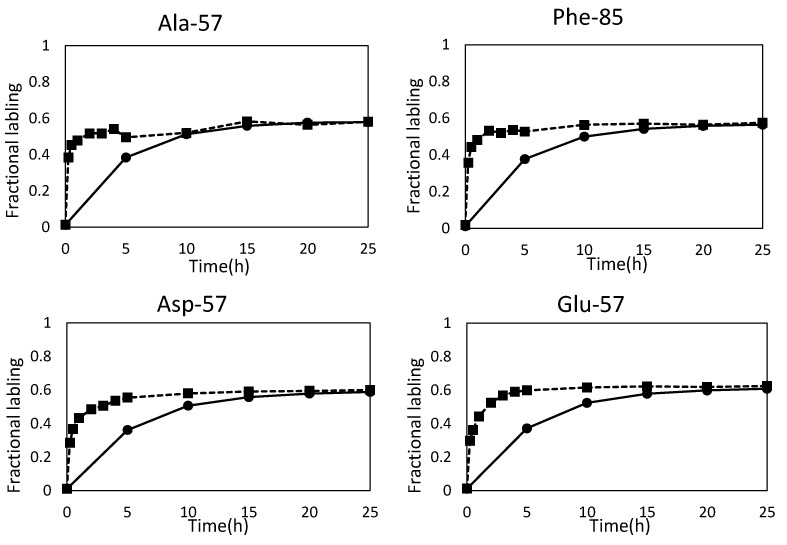
Time course of ^13^C enrichment of proteinogenic and free amino acids. Fractional labeling of proteinogenic amino acids (PAAs, Closed circle) and free amino acids (FAAs, Closed square) are represented. Fractional labeling is a ratio of labeled carbons in the metabolite. Other data are shown in Figure S2.

In this study, FAAs-based MFA was developed by using *E. coli* MG1655 strain. *E. coli* is one of the most widely-used model organisms in the fields of metabolic engineering and systems biology. In order to compare the PAAs- and FAAs-based methods, ^13^C enrichment of PAAs and FAAs were determined for *E. coli* cells obtained from a same culture. The continuous culture of *E. coli* cells was performed at a dilution rate of 0.2 h^−1^ using a synthetic medium containing glucose. After 8 residence time, the metabolic steady state was confirmed from constant levels of OD_600_, concentration of O_2_ and CO_2_ in exhausted gas and concentration of organic acids in medium (Figure S1). Specific uptake rate of glucose and the specific production rate of acetate, formate, lactate, and ethanol in the metabolic steady state were 3.41 ± 0.01, 1.13 ± 0.06, 0.98 ± 0.05, 0.09 ± 0.01, and 0.01 ± 0.01 mmol/g-DCW/h, respectively. After attaining the metabolic steady state, the carbon source in the feeding medium was changed from 100% natural glucose to a mixture of glucose containing 1.0% non-labeled glucose, 49.2% [1-^13^C] glucose and 49.8% [U-^13^C] glucose. The *E. coli* cells were repeatedly collected from the culture, and the ^13^C enrichment of PAAs and FAAs were determined using the GC-MS analysis. Time course of ^13^C enrichment of representative PAAs and FAAs are shown in Figure 1. While an isotopic steady state was attained at 25 h (5 residence time) after the start of ^13^C labeling for the case of PAAs, the ^13^C enrichment of FAAs reached plateau in 10 h (2 residence time). This result confirms that the FAAs-based method can reduce labeling time for MFA. Although the faster turnover rate for FAAs is preferable for an MFA study of batch and fed-batch culture [12,13,14,15], it remains unclear whether a reliable result can be produced by FAAs-based MFA. It is not also obvious which FAAs are reproducibly observed from *E. coli* cells in various culture conditions, and whether a precise metabolic flux can be estimated by using the observed amino acids.

### 2.2. Metabolic Flux Analysis

**Figure 2:**
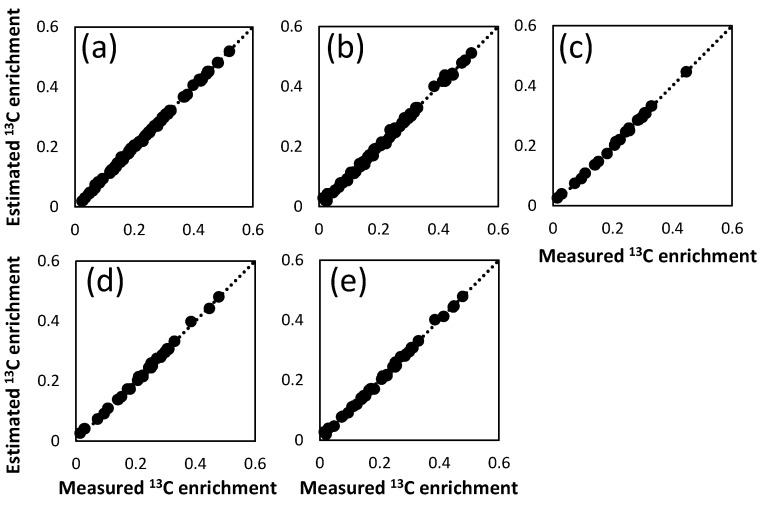
Comparison of measured and estimated ^13^C enrichment of amino acids. The X axis and Y axis indicate measured and estimated ^13^C enrichment, respectively, of the (**a**) PAAs_fullset; (**b**) FAAs_fullset; (**c**) FAAs_Glu+Asp; (**d**) FAAs_Glu+Asp+Ala; and (**e**) FAAs_Glu+Asp+Ala+Phe.

**Figure 3:**
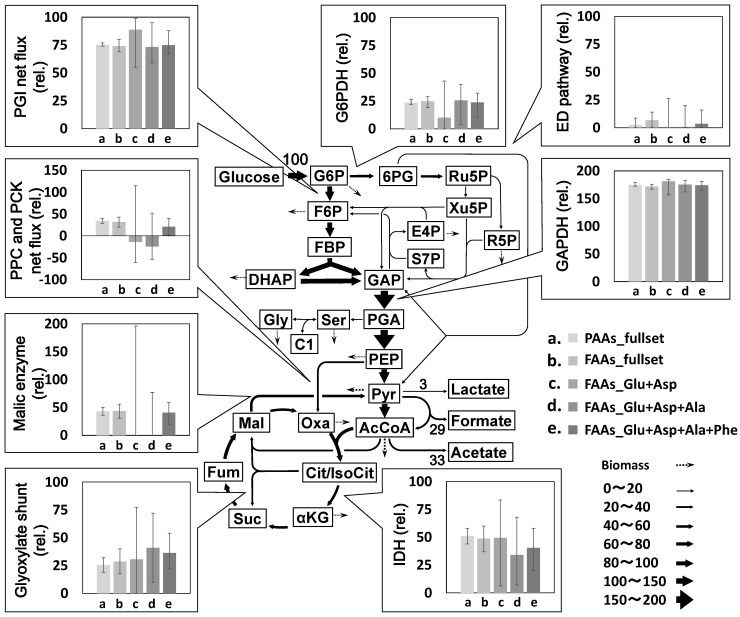
Comparison of metabolic flux distributions estimated using different datasets. Flux values are normalized to a glucose uptake rate of 100. Fluxes and 95% confidence intervals in representative reactions are shown. Full data is available from Tables S1-1, S2-1, S3-1, S4-1 and S5-1.

Performance of FAAs-based MFA method was investigated by comparison with that of PAAs-based method. For this purpose, a reference metabolic flux distribution was determined by the PAAs-based MFA. From the *E. coli* cells obtained at 25 h after the start of the tracer experiment, PAAs were prepared via acid hydrolysis of the proteins. The GC-MS analysis of the derivatized samples successfully determined the ^13^C enrichment of 25 fragments derived from a total of 11 amino acids (Table 1). Using all of the ^13^C enrichment data (PAAs_fullset), a reference metabolic flux distribution was estimated by a non-linear fitting to a metabolic model (Tables S1-1 and S1-2). The estimated and measured ^13^C enrichment were almost similar to each other, indicating that a reasonable flux distribution was estimated from the best-fitted results (Figure 2a). Figure 3 shows the estimated fluxes in their representative reactions. The results of PAAs_fullset (a in Figure 3) show that 75% of glucose is catabolized via the glycolysis (PGI net flux) and 25% via the pentose phosphate (PP) pathway (G6PDH flux). The Entner-Doudoroff (ED) pathway is inactive in this condition. While a significant amount of pyruvate (Pyr) and acetyl-CoA (AcCoA) are secreted to the medium as formate and acetate, the remaining carbon flows into the TCA cycle. The flux distribution is essentially comparable to a previously reported result [17]. In this study, 95% confidence intervals of each flux were estimated by the grid search method (represented as error bars in Figure 3). The 95% confidence intervals of glycolysis/PP pathway branch point were deduced to be ± 3, and the flux intervals of upper glycolysis, PP pathway and ED pathway were estimated to be within ± 6. Relatively large 95% confidence intervals (± 13) were observed for fluxes in lower glycolysis, TCA cycle, glyoxylate shunt and anaplerosis.

**Table 1:** Data points used for metabolic flux analysis and residual sum of squares (RSS) of best-fitted results.

	Number of independent measurements (=*n*)	Number of metabolites used for fitting	Number of fragments used for fitting	*n*-*p* ^1^	RSS	RSS/(*n*-*p*)
PAAs_fullset	92	11	25	71	0.0013	0.00002
FAAs_fullset	66	9	19	45	0.0037	0.00008
FAAs_Glu+Asp	25	2	7	9	0.0007	0.00017
FAAs_Glu+Asp+Ala	30	3	9	19	0.0012	0.00014
FAAs_Glu+Asp+Ala+Phe	40	4	11	4	0.0015	0.00008

^1^ *p*: Degree of freedom of metabolic model (=21).

The arrow width represents the flux levels estimated by using the reference (PAAs_fullset) dataset. Abbreviations: PGI: phosphoglucose isomerase, PPC: phosphoenolpyruvate carboxylase, PCK: phosphoenolpyruvate carboxykinase, G6PDH: glucose 6-phosphate dehydrogenase, GAPDH: glyceraldehyde 3-phosphate dehydrogenase, IDH: isocitrate dehydrogenase, G6P: glucose 6-phosphate, F6P: fructose 6-phosphate, FBP: fructose 1,6-bisphosphate, DHAP: dihydroxyacetone phosphate, GAP: glyceraldehyde 3-phosphate, PGA: 3-phosphoglycerate, PEP: phosphoenolpyruvate, Pyr: pyruvate, AcCoA: acetyl-coenzyme A, Cit: citrate, αKG: α-ketoglutarate, Suc: succinate, Fum: fumarate, Mal: malate, Oxa: oxaloacetate, 6PG: 6-phosphogluconate, Ru5P: ribulose 5-phosphate, R5P: ribose 5-phosphate, Xu5P: xylulose 5-phosphate, S7P: sedoheptulose 7-phosphate, E4P: erythrose 4-phosphate, Ala: alanine, Phe: phenylalanine, Asp: aspartate, Glu: glutamate.

From the same *E. coli* culture, a flux distribution was estimated using FAAs. Intracellular FAAs were extracted from the *E. coli* cells at 15, 20, and 25 h after the start of the tracer labeling. The ^13^C enrichment of 19 fragments derived from 11 amino acids was determined with signal to noise ratio > 10 by GC-MS analysis (FAAs_fullset). A flux distribution was successfully estimated by a non-linear fitting of the metabolic model to the mean ^13^C enrichment data (Figure 2b, Tables S2-1 and S2-2). As shown in Figure 3, the metabolic flux distribution estimated from FAAs data (FAAs_fullset, b in Figure 3) was quite similar to the reference data determined by PAAs-based MFA (PAAs_fullset, a in Figure 3).

An investigation of the continuous culture of *E. coli* using a simplified metabolic model demonstrated that PAAs- and FAAs-based MFA methods can produce compatible flux distributions [17]. The MFA performed in this study using the generally accepted metabolic model confirmed that essentially identical flux distributions are estimated from the ^13^C enrichment data of PAAs (PAAs_fullset) and FAAs (FAAs_fullset), with the 95% confidence intervals of the two sets overlapping each other (Figure 2 and Figure 3).

A comparison of the confidence intervals also reveals that a flux distribution estimated from the FAAs_fullset shows wider confidence intervals (Figure 3). It is because the number of measurable fragments, amino acids and independent measurements of the FAAs_fullset (*n* = 66) is significantly less than that for the PAAs_fullset (*n* = 92, Table 1). The smaller amount of ^13^C enrichment data cannot be overlooked in the FAAs-based method when directly analyzing FAAs with low concentrations.

### 2.3. Combination of Amino Acids for Reliable FAAs-Based MFA

**Figure 4:**
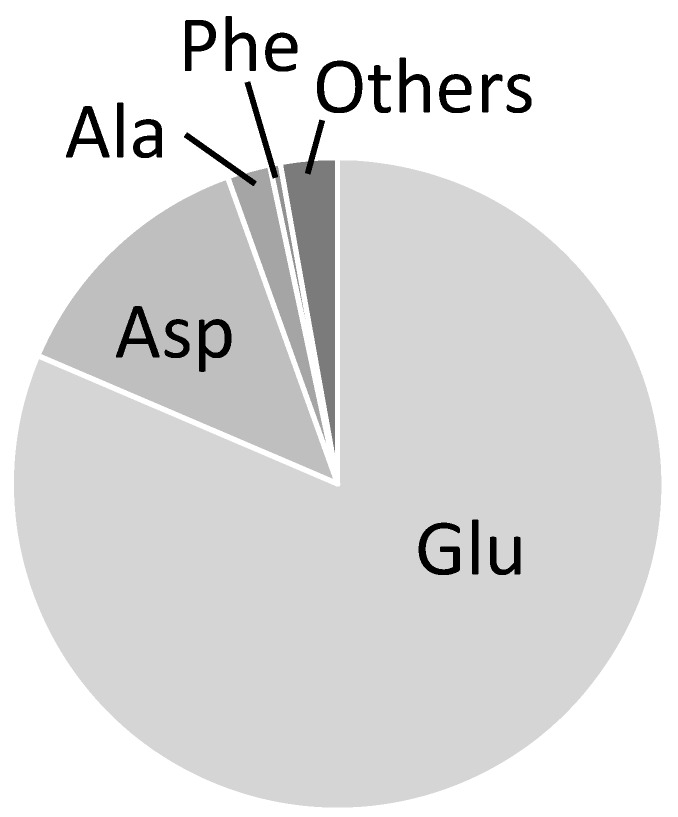
Signal intensities of free amino acids determined by GC-MS. Total signal intensities of Glu ([M-57], [M-85], [M-159]), Asp ([M-57], [M-85], [M-159], [f302]), Ala ([Ala-57], [Ala-85]) and Phe ([M-85], [f302]) are shown in the figure.

Measurable FAAs should vary among the FAA-based MFA experiments since the pool sizes of intracellular free metabolites depend on cellular metabolic conditions. For instance, it was reported that the pool size of FAAs in *E. coli* ranged from 2 × 10^−2^ to 80 µmol/g-DCW [18]. Indeed, previous studies demonstrated that distinct sets of FAAs were observed in the FAAs-based MFA at different culture conditions (Table 2) [14,17,19]. For the amino acids shown in Table 2, Glu, Asp, Ala, and Phe are the four amino acids commonly observed in the four studies, suggesting that these amino acids are likely to be measurable from *E. coli* cells at various culture conditions (Table 2). These amino acids were also commonly observed in various single gene knock-out *E. coli* strains [20]. In the GC-MS data obtained in this study, the signal of glutamate is rather intense than for the other amino acids (Figure 4). The second, third and fourth most intense signals are from Asp, Ala, and Phe, respectively. A similar tendency was also reported [20], indicating that glutamate is a useful amino acid to investigate for the ^13^C enrichment of α-ketoglutarate (αKG) in FAAs-based MFA.

In order to test the performance of FAA-based MFA experiment using Glu, Asp, Ala, and Phe, flux distributions were estimated using the ^13^C enrichment data of 7 fragments of glutamate and aspartate (FAAs_Glu+Asp), 9 fragments of glutamate, aspartate, and alanine (FAAs_Glu+Asp+Ala), and 11 fragments of glutamate, aspartate, alanine and phenylalanine (FAAs_Glu+Asp+Ala+Phe), respectively (Table 1). The results obtained from each dataset are shown in Figure 2c–e and Figure 3, Tables S3-1, S3-2, S4-1, S4-2, S5-1 and S5-2. The comparison of the results with the reference data (PAAs_fullset) revealed that, in the case of the FAAs_Glu+Asp (c in Figure 3) and FAAs_Glu+Asp+Ala (d in Figure 3) datasets, very large 95% confidence intervals are observed especially for flux of the malic enzyme reaction and phosphoenolpyruvate carboxylase (PPC) and phosphoenolpyruvate carboxykinase (PCK) net flux (Figure 3). On the other hand, the FAAs_Glu+Asp+Ala+Phe (e in Figure 3) dataset produced a rather precise metabolic flux distribution with narrower confidence intervals. For example, the 95% confidence interval of PPC and PCK net flux and malic enzyme flux determined by FAAs_Glu+Asp+Ala+Phe were 38% and 51% of that of FAAs_Glu+Asp+Ala, respectively. This result indicates that Glu, Asp, Ala, and Phe constitute a practical minimal set for FAAs-based MFA.

**Table 2:** Free amino acids measured in ^13^C-metabolic flux analysis studies using *E. coli*.

	This study	Mori *et al.* (2011) [17]	Toya *et al.* (2010) [19]	Iwatani *et al.* (2007) [14]
*Experimental conditions*				
Analysis	GC-MS	GC-MS	CE-TOFMS	LC-MS/MS
Culture	Chemostat culture	Chemostat culture	Batch culture	Fed-batch culture
***Amino acids***				
Alanine	+	+	+	+
Valine	+	-	+	+
Leucine	+	+	+	-
Isoleucine	-	-	+	-
Lysine	-	-	+	-
Aspartate	+	+	+	+
Asparagine	-	-	-	+
Threonine	+	-	+	+
Methionine	-	-	-	-
Glutamate	+	+	+	+
Glutamine	-	-	-	+
Arginine	-	-	+	-
Proline	-	-	+	-
Glycine	+	-	+	+
Serine	-	+	+	+
Cysteine	-	-	-	-
Histidine	-	-	+	-
Tyrosine	+	-	+	+
Phenylalanine	+	+	+	+
Tryptophan	-	-	-	-

+: detected; -: not detected.

According to the study of flux ratio analysis reported by Fischer and Sauer [21], labeling patterns of ^13^C enrichment of Pyr and oxaloacetate (Oxa) are essential to elucidate the branch ratio of glycolysis, PP pathway and ED pathway or TCA cycle and glyoxylate shunt. This indicates that ^13^C-labeling of Ala and Asp synthesized from Pyr and Oxa, respectively, is necessary to estimate flux in various flux distributions in the central carbon metabolism. The metabolic flux analysis using FAAs_Glu+Asp and FAAs_Glu+Asp+Ala shows that, whereas levels of PGI and GAPDH net fluxes are similar to that of the reference data (PAA_fullset), large confidence intervals are observed than for the case of the other metabolic fluxes (Figure 3). The result indicates that, in addition to the ^13^C enrichment information of αKG, Oxa, and Pyr derived from Glu, Asp, and Ala, respectively, an analysis of other amino acids is required for a more precise estimation of the metabolic flux distribution. The confidence intervals of metabolic flux analysis were drastically improved by employing the FAAs_Glu+Asp+Ala+Phe dataset, indicating a requirement for ^13^C enrichment information of PEP and E4P in Phe (Figure 3). Indeed, it has been reported that levels of metabolic flux in anaplerotic pathways can be well estimated from PEP in addition to Pyr, Oxa, and αKG [21].

These results suggest that a combination of Ala, Asp, Glu, and Phe synthesized from Pyr, Oxa, αKG, PEP, and E4P is a requirement for FAAs-based MFA using [1-^13^C] and [U-^13^C] glucose. This mixture of ^13^C-labeled glucose was generally used in various MFA studies [1,12,13,14,17,19,21,22]. Since there are significant fluxes in each reaction in the metabolic network shown in Figure 3, the four amino acids should contain enough information to determine metabolic fluxes in other culture conditions. The additional analysis of other amino acids (Table 2) is expected to improve the confidence intervals of the estimated metabolic fluxes. The findings in this study are most applicable for GC-MS analysis and further experiments would be needed to show that this knowledge is also applicable to MFA based on intermediate metabolites using LC-MS. Although additional confirmations are required, our approach also would be available to other microorganisms with similar metabolic pathway, such as *Bacillus subtilis* and *Corynebacterium glutamicum*.

## 3. Experimental Section

### 3.1. Strain and Medium

*Escherichia coli* K-12 MG1655 strain was cultured in M9 medium consisting of 17.1 g/L Na_2_HPO_4_ • 12H_2_O, 3 g/L KH_2_PO_4_, 0.5 g/L NaCl, 2 g/L NH_4_Cl, 123 mg/L MgSO_4_ • 7H_2_O, 2.78 mg/L FeSO_4_ • 7H_2_O, 14.7 mg/L CaCl_2_ • 2H_2_O, 10 mg/L thiamine-hydrochloride and 5 g/L glucose. Adecanol (1 × 10^−3^% *w*/*w*) was added as an antiform in the continuous culture. [1-^13^C] glucose (98%–99%) and [U-^13^C] glucose (99%) were purchased from Cambridge Isotope Laboratories (Andover, MA, USA).

### 3.2. Culture Condition

A frozen stock of *E. coli* cells was inoculated in 40 mL M9 medium and incubated for 14 h at 37 °C with reciprocal shaking. Continuous culture was performed in a 1 L bioreactor (ABLE, Tokyo, Japan) equipped with temperature, pH, dissolved oxygen, and exhaust gas (O_2_ and CO_2_) sensors. The working volume and aeration rate were 400 mL and 400 mL/min, respectively. The temperature was controlled at 37 °C, and pH was set at 7.0 using an NH_3_ solution. Five hours after inoculation, the continuous culture was started at a dilution rate of 0.2 h^−1^. The feeding medium containing natural glucose was replaced with one containing ^13^C-labeled glucose after the continuous culture reached a metabolic steady state. Labeling of glucose in the medium was determined by gas chromatography–mass spectrometry (GC-MS) analysis by a previously described method [22].

### 3.3. Off-Line Measurements

Levels of OD_600_ were determined using a spectrophotometer (UVmini-1240, Shimadzu, Kyoto, Japan). A glucose sensor (Bioanalyzer BF-5, Oji Scientific Instruments, Hyogo, Japan) was used for glucose analysis. Ethanol concentration was measured using a gas chromatograph (Agilent 7890A GC; Agilent Technologies, Santa Clara, USA) operated at the following conditions; column, Stabilwax 60 m × 0.32 mm ID × 1 μm (Restek, Bellefonte, USA); carrier gas, helium; flow rate, 6.5 mL/min; injection volume, 1 μL; split ratio, 1:10; oven temperature, 70 °C for 3 min and raised at 10 °C/min; FID detector temperature, 250 °C. The concentrations of organic acids (lactate, formate, acetate, succinate and fumarate) were determined by a high-performance liquid chromatography (HPLC) system (Prominence; SHIMADZU, Kyoto, Japan, column, Oapak-A; 7.8 mm ID × 30 cm (TOSOH, Tokyo, Japan); column temperature, 40 °C; mobile phase, 0.75 mM H_2_SO_4_ in water; flow rate, 0.8 mL/min; detection, UV 210 nm).

### 3.4. Sample Preparation for GC-MS Analysis

For analysis of the PAAs, 10 mL broth culture was taken directly from the reactor and centrifuged at 9800× *g* for 10 min at 4 °C. The cell pellet was washed twice with 0.9% NaCl and hydrolyzed in 2 mL 6 *N* HCl at 105 °C for 18 h. After filtration (Cosmonice filter W, pore size 0.45 µm, Filter diameter 13 mm, Nacalai Tesque, Kyoto, Japan), 10 µL of internal standard (600 µM cycloleucine) was added to 10 µL of the hydrolysate and evaporated to dryness. The dried residue was dissolved in 50 µL acetonitrile and 50 µL N-(tert-butyldimethylsilyl)-N-methyl-trifluoroacetamide containing 1% *tert*-butyldimethylchlorosilane, and incubated at 105 °C for 1 h. After 1 h cooling, a supernatant was injected for GC-MS analysis. Intercellular FAAs were extracted by the following procedure. Cells were collected by filtration of 5 mL of culture broth (PTFE type membrane, 0.5 µm pore size, diameter 90 mm, ADVANTEC, Tokyo,Japan) [23,24]. Cells on the filter were immediately immersed in 1.6 mL methanol (−80 °C) and preserved at −80 °C. After the frozen cell sample was suspended in 1.6 mL of chloroform (−30 °C), 630 µL Milli-Q water (4 °C) and 10 µL 600 µM cycloleucine dissolved in water were added. After vortexing and sonication for 1 min, the mixture was centrifuged at 3700× *g* for 40 min at 4 °C. A 2 mL aqueous layer was evaporated to dryness (Speed Vac, Thermo Schientific, Waltham, Japan).

### 3.5. GC-MS Analysis of PAAs and FAAs

The mass isotopic distributions of four type of ion clusters at mass to charge (*m*/*z*) rations of [M-57], [M-85], [M-159], and [f302] derived from each amino acid were determined by using GC-MS (Agilent 7890A GC and 5975C Mass Selective Detector (Agilent Technologies, Santa Clara, USA); column, DB-5MS+DG; 30 m × 0.25 mm ID × 0.25 μm; (Agilent Technologies, Santa Clara, USA); carrier gas, Helium; flow rate, 1.0 mL/min; detection mode, selected ion monitoring; ion source temperature, 230 °C; electron impact ionization, 70 eV). PAAs were analyzed under the following conditions: injection volume, 1 μL; split ratio, 1:10; oven temperature, 150 °C for 2 min, increased by 3 °C/min to 270 °C, then increased at a rate of 10 °C/min to 300 °C, and maintained at that temperature for 5 min [17]. For the analysis of FAAs, splitless mode was employed to measure small amount of FAAs. Detailed conditions are as follows: injection volume, 1 μL; splitless mode; oven temperature, 60 °C for 2 min, increased by 20 °C/min to 150 °C, then increased at a rate of 3 °C/min to 270 °C, further increased to 300 °C at 10 °C/min and maintained at that temperature for 5 min.

### 3.6. Metabolic Flux Analysis

A metabolic model including glycolysis, the tricarboxylic acid (TCA) cycle, glyoxylate shunt, anaplerosis, the pentose phosphate (PP) pathway, the Entner-Doudoroff (ED) pathway, CO_2_ exchange and C1 metabolism was employed for flux analysis [21]. The number of free flux or degree of freedom of the model was 21 (Table S1). The fluxes for biomass synthesis of E. coli were calculated from the precursor requirement [25]. The effect of naturally occurring isotopes was removed from the raw mass spectrometry data to obtain corrected ^13^C labeling patterns of the carbons in the amino acids [26]. The fragments of amino acids were chosen according to the report of Antoniewicz *et al.* [27]. Additionally, the appropriate fragments were carefully screened by comparing the natural and theoretical isotope abundance ratios. The calculation of fluxes was performed using the in-house software OpenMebius [28], which is a flux estimation tool using the elementary metabolite units (EMU) framework in Matlab 2011b [29]. In short, the fluxes were estimated by minimizing the residual sum of squares (RSS) between the experimentally measured ^13^C enrichment and the estimated value using the *fmincon* optimization solver in the Matlab toolbox. The optimizing function is described as:

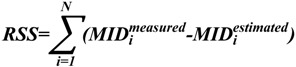

where 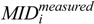 is the mass isotopomer distribution (MID) of the ith measured metabolite, 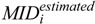 is the estimated MID of the corresponding metabolite, and N is the number of metabolites used for flux estimation. Optimization was started from 20 sets of random flux distributions. Confidence intervals were calculated by a grid search method as described previously [30,31,32]. The metabolic flux of reaction *r* is fixed to *v_opt,r_* + *d* and the objective function is re-optimized. Here, *v_opt,r_* is the optimized metabolic flux of reaction *r* and *d* is the perturbation level. The procedure is iterated with increased or decreased *d*. The range of fixed metabolic flux whose RSS is less than the threshold level is the confidence interval. The threshold level is determined by:

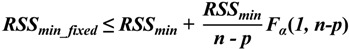

where *RSS_min_fixed_* is the minimized RSS with one fixed flux, *RSS_min_* is the original minimized RSS, *n* is the number of independent data points used in the fitting, *p* is the degrees of freedom in the original flux fit, *F* is the *F*-distribution, and α is the confidence level.

## 4. Conclusions

In this study, a performance of the metabolic flux analysis using free amino acids (FAAs) was directly compared with that using proteinogenic amino acids (PAAs). An estimation of 95% confidence intervals for the first time demonstrated that the FAAs-based MFA has essentially identical reliability with that obtained from the PAAs-based method (Figure 3). It was also experimentally demonstrated that the amino acids derived from Pyr, Oxa, αKG, PEP, and E4P are at least required for FAAs-based MFA. The minimal set of amino acids for precise MFA can be a practical guide for the design of MFA experiments using FAAs.

## Supplementary Files

Supplementary File 1 Supplementary File (PDF, 563 KB)
