# Supplementary material for: Reliable Metabolic Flux Estimation in Escherichia coli Central Carbon Metabolism Using Intracellular Free Amino Acids
Source: Metabolites. 2014 May 30;4(2):408–20. doi: 10.3390/metabo4020408 (PMC4101513; doi:10.3390/metabo4020408)
Supplement: Supplementary File 1 — Supplementary File (PDF, 563 KB) [file metabolites-04-00408-s001.pdf]

Supplementary File

Table S1. Metabolic model.

Flux number	Reaction	Carbon transitions
r1	Glucose --> G6P	ABCDEF --> ABCDEF
r2	G6P --> F6P	ABCDEF --> ABCDEF
r3	F6P --> G6P	ABCDEF --> ABCDEF
r4	F6P --> FBP	ABCDEF --> ABCDEF
r5	FBP --> DHAP + GAP	ABCDEF --> CBA + DEF
r6	DHAP + GAP --> FBP	CBA + DEF --> ABCDEF
r7	DHAP --> GAP	ABC --> ABC
r8	GAP --> DHAP	ABC --> ABC
r9	GAP --> PGA	ABC --> ABC
r10	PGA --> GAP	ABC --> ABC
r11	PGA --> PEP	ABC --> ABC
r12	PEP --> PGA	ABC --> ABC
r13	PEP --> Pyr	ABC --> ABC
r14	Pyr --> AcCoA + CO_2_in	ABC --> BC + A
r15	AcCoA + Oxa --> IsoCit	AB + CDEF --> FEDBAC
r16	IsoCit -->αKG + CO_2_in	ABCDEF --> ABCDE + F
r17	αKG --> Suc + CO_2_in	ABCDE --> BCDE + A
r18	Suc --> Fum	ABCD --> ABCD
r19	Fum --> Suc	ABCD --> ABCD
r20	Fum --> Mal	ABCD --> ABCD
r21	Mal --> Fum	ABCD --> ABCD
r22	Mal --> Oxa	ABCD --> ABCD
r23	Oxa --> Mal	ABCD --> ABCD
r24	IsoCit + AcCoA --> Mal + Suc	ABCDEF + GH --> ABHG + FCDE
r25	PEP + CO2_in --> Oxa	ABC + D --> ABCD
r26	Oxa --> PEP + CO_2_in	ABCD --> ABC + D
r27	Mal --> Pyr + CO_2_in	ABCD --> ABC + D
r28	G6P --> 6PG	ABCDEF --> ABCDEF
r29	6PG --> Ru5P + CO_2_in	ABCDEF --> BCDEF + A
r30	Ru5P --> R5P	ABCDE --> ABCDE
r31	R5P --> Ru5P	ABCDE --> ABCDE
r32	Ru5P --> Xu5P	ABCDE --> ABCDE
r33	Xu5P --> Ru5P	ABCDE --> ABCDE
r34	R5P + Xu5P --> S7P + GAP	ABCDE + FGHIJ --> FGABCDE + HIJ
r35	GAP + S7P --> Xu5P + R5P	HIJ + FGABCDE --> FGHIJ + ABCDE
r36	GAP + S7P --> F6P + E4P	ABC + DEFGHIJ --> DEFABC + GHIJ
r37	E4P + F6P --> S7P + GAP	GHIJ + DEFABC --> DEFGHIJ + ABC
r38	E4P + Xu5P --> F6P + GAP	ABCD + EFGHI --> EFABCD + GHI
r39	GAP + F6P --> Xu5P + E4P	GHI + EFABCD --> EFGHI + ABCD
r40	6PG --> Pyr + GAP	ABCDEF --> ABC + DEF
r41	PGA --> Ser	ABC --> ABC
r42	Ser --> Gly + THF_in	ABC --> AB + C
r43	Gly + THF_in --> Ser	AB + C --> ABC

Table S1. *Cont.*

Flux number	Reaction	Carbon transitions
r44	G6P --> [Biomass]	
r45	F6P --> [Biomass]	
r46	DHAP --> [Biomass]	
r47	Ser --> [Biomass]	
r48	Gly --> [Biomass]	
r49	PEP --> [Biomass]	
r50	Pyr --> [Biomass]	
r51	AcCoA --> [Biomass]	
r52	αKG --> [Biomass]	
r53	Oxa --> [Biomass]	
r54	R5P --> [Biomass]	
r55	E4P --> [Biomass]	
r56	Pyr --> [Lactate_ex]	
r57	Pyr --> AcCoA + [Formate_ex]	ABC --> BC + A
r58	AcCoA --> [Acetate_ex]	
R59	AcCoA --> [EtOH_ex]	
r60	CO2_in --> [CO2_ex]	A --> A
r61	CO2 --> CO2_in	A --> A
r62	THF_in --> [THF_ex]	A --> A
r63	THF --> THF_in	A --> A

Figure S1. Fermentation profile. (**a**) The time course of optical density at 600 nm (Closed diamond) and the concentration of remained glucose (Closed square) and produced ethanol (Closed triangle) are shown. The concentration of glucose was zero constantly, indicating that glucose-limited chemostat culture was achieved. (**b**) The time course of organic acids concentration are shown. Closed circle, square, triangle and diamond indicates pyruvate, lactate, formate and acetate, respectively. The concentration was constant after 30 h from the start of culture, indicating that quasi metabolic steady state was assumable.

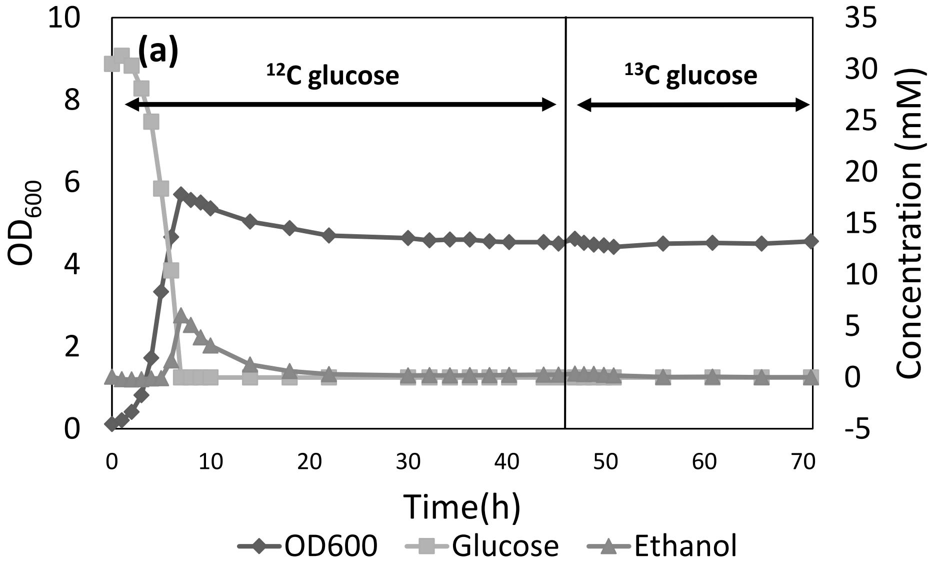

Figure S1. *Cont.*

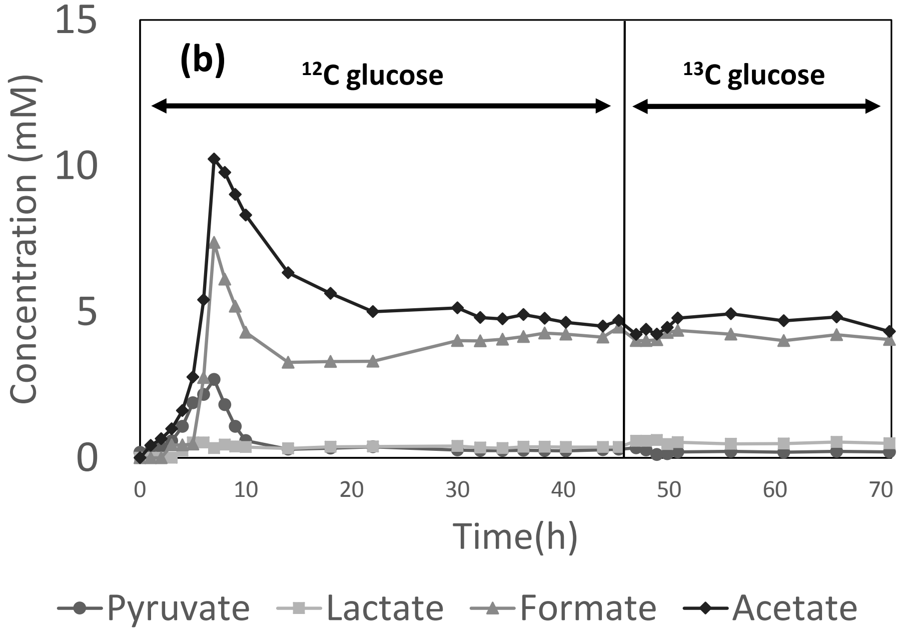

Figure S2. Time course of ^{13}C enrichment of PAAs and FAAs. Fractional labeling of PAAs (Closed circle) and FAAs (Closed square) after the start of tracer feeding is shown in the figure.

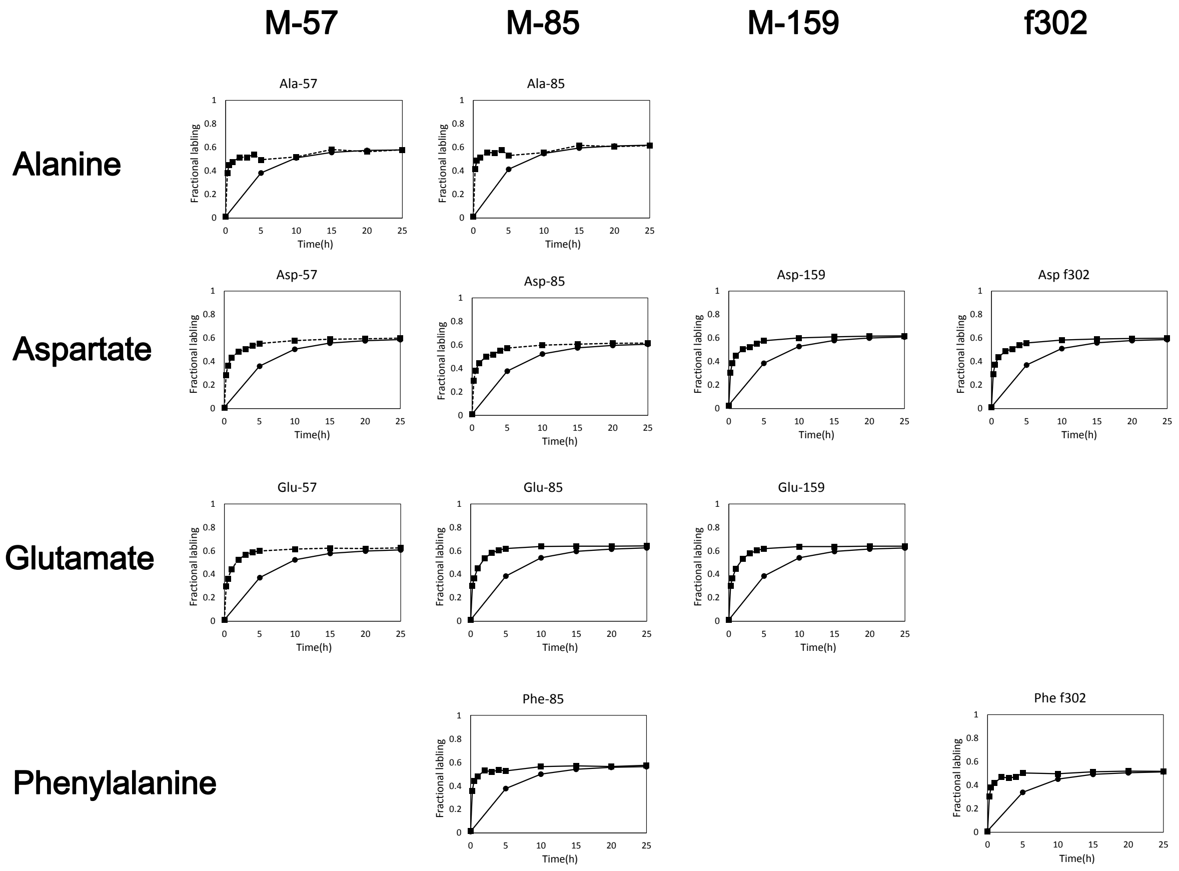

Figure S2. *Cont.*

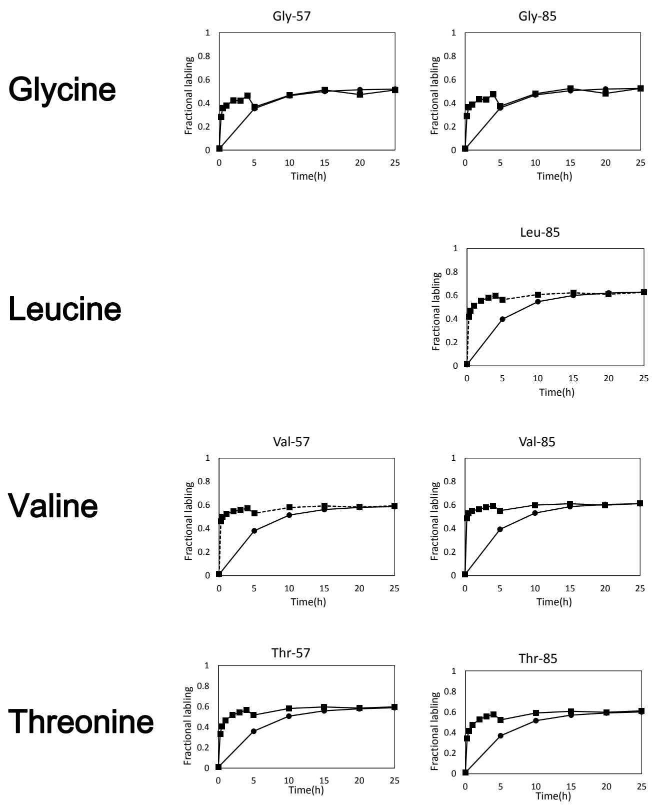

Supplementary Data 1

Results of metabolic flux analysis based on the PAAs_fullset

Table S1-1. Estimated flux distribution and its 95% confidence interval.

Reaction	Lower	Best fit	Upper
G6P <-> F6P	74	75	77
F6P -> FBP	83	85	86
FBP <-> DHAP + GAP	83	85	86
DHAP <-> GAP	82	84	85
GAP <-> PGA	172	175	176
PGA <-> PEP	164	166	167
PEP -> Pyr	122	129	136
Pyr -> AcCoA	122	128	136
AcCoA + Oxa -> Cit	75	77	78
Cit ->αKG	44	51	58
αKG -> Suc	38	45	52
Suc <-> Fum	69	70	71
Fum <-> Mal	69	70	71
Mal <-> Oxa	48	53	57
Glyoxylate shunt	19	25	32
PEP + CO_2 <-> Oxa	29	34	40

Table S1-1. *Cont.*

Reaction	Lower	Best fit	Upper
Mal -> Pyr	36	43	50
G6P -> 6PG	22	24	25
6PG -> Ru5P	18	21	24
Ru5P <-> R5P	10	11	12
Ru5P <-> Xu5P	8	10	12
R5P + Xu5P <-> S7P + GAP	5	6	7
GAP + S7P <-> E4P +F6P	5	6	7
E4P + Xu5P <-> GAP + F6P	3	4	5
ED pathway	0	3	6

Table S1-2. Measured and estimated MID of PAAs_fullset.

Name	Mass isotopomer	Measured MID	Estimated MID
PAA_Ala-57	M	0.213	0.215
	M+1	0.228	0.219
	M+2	0.160	0.160
	M+3	0.398	0.406
PAA_Ala-85	M	0.248	0.249
	M+1	0.269	0.270
	M+2	0.483	0.481
PAA_Asp-57	M	0.083	0.083
	M+1	0.181	0.178
	M+2	0.245	0.245
	M+3	0.276	0.277
	M+4	0.215	0.216
PAA_Asp-85	M	0.120	0.121
	M+1	0.251	0.248
	M+2	0.318	0.321
	M+3	0.310	0.310
PAA_Asp-159	M	0.119	0.121
	M+1	0.247	0.248
	M+2	0.320	0.321
	M+3	0.313	0.310
PAA_Asp302	M	0.267	0.270
	M+1	0.290	0.287
	M+2	0.443	0.443
PAA_Glu-57	M	0.033	0.030
	M+1	0.095	0.094
	M+2	0.205	0.205
	M+3	0.283	0.283
	M+4	0.237	0.238
	M+5	0.148	0.149

Table S1-2. *Cont.*

Name	Mass isotopomer	Measured MID	Estimated MID
PAA_Glu-85	M	0.048	0.045
	M+1	0.138	0.139
	M+2	0.294	0.299
	M+3	0.300	0.298
	M+4	0.220	0.219
PAA_Glu-159	M	0.049	0.045
	M+1	0.139	0.139
	M+2	0.295	0.299
	M+3	0.298	0.298
	M+4	0.219	0.219
PAA_Phe-57	M	0.023	0.020
	M+1	0.045	0.043
	M+2	0.082	0.080
	M+3	0.120	0.118
	M+4	0.139	0.136
	M+5	0.171	0.172
	M+6	0.143	0.147
	M+7	0.123	0.125
	M+8	0.086	0.087
	M+9	0.067	0.074
PAA_Phe-85	M	0.026	0.021
	M+1	0.049	0.048
	M+2	0.117	0.117
	M+3	0.139	0.134
	M+4	0.169	0.171
	M+5	0.142	0.143
	M+6	0.162	0.165
	M+7	0.118	0.117
	M+8	0.078	0.084
PAA_Phe302	M	0.424	0.420
	M+1	0.129	0.128
	M+2	0.447	0.451
PAA_Gly-57	M	0.419	0.425
	M+1	0.130	0.123
	M+2	0.451	0.452
PAA_Gly-85	M	0.480	0.481
	M+1	0.520	0.519
PAA_Ile-85	M	0.034	0.030
	M+1	0.092	0.094
	M+2	0.199	0.205
	M+3	0.283	0.283
	M+4	0.239	0.238
	M+5	0.153	0.149

Table S1-2. *Cont.*

Name	Mass isotopomer	Measured MID	Estimated MID
PAA_Ile-159	M	0.034	0.030
	M+1	0.093	0.094
	M+2	0.202	0.205
	M+3	0.285	0.283
	M+4	0.239	0.238
	M+5	0.147	0.149
PAA_Leu-85	M	0.024	0.020
	M+1	0.084	0.085
	M+2	0.186	0.191
	M+3	0.295	0.296
	M+4	0.248	0.251
	M+5	0.162	0.158
PAA_Ser-57	M	0.189	0.189
	M+1	0.278	0.270
	M+2	0.155	0.166
	M+3	0.378	0.375
PAA_Ser-85	M	0.206	0.207
	M+1	0.366	0.367
	M+2	0.429	0.426
PAA_Ser-159	M	0.208	0.207
	M+1	0.369	0.367
	M+2	0.423	0.426
PAA_Val-57	M	0.059	0.054
	M+1	0.117	0.113
	M+2	0.204	0.202
	M+3	0.250	0.250
	M+4	0.182	0.187
	M+5	0.188	0.195
PAA_Val-85	M	0.067	0.062
	M+1	0.134	0.135
	M+2	0.310	0.313
	M+3	0.258	0.260
	M+4	0.231	0.231
PAA_Tyr302	M	0.424	0.420
	M+1	0.129	0.128
	M+2	0.448	0.451
PAA_Thr-57	M	0.082	0.083
	M+1	0.180	0.178
	M+2	0.244	0.245
	M+3	0.278	0.277
	M+4	0.217	0.216
PAA_Thr-85	M	0.121	0.121
	M+1	0.251	0.248
	M+2	0.323	0.321
	M+3	0.305	0.310

Supplementary Data 2

Results of metabolic flux analysis based on the FAAs_fullset

Table S2-1. Estimated flux distribution and its 95% confidence interval.

Reaction	Lower	Best fit	Upper
G6P <-> F6P	69	74	80
F6P -> FBP	76	81	86
FBP <-> DHAP + GAP	76	81	86
DHAP <-> GAP	76	81	85
GAP <-> PGA	167	172	176
PGA <-> PEP	158	163	167
PEP -> Pyr	122	128	142
Pyr -> AcCoA	116	133	145
AcCoA + Oxa -> Cit	75	78	81
Cit ->αKG	37	49	60
αKG -> Suc	30	43	53
Suc <-> Fum	68	71	75
Fum <-> Mal	68	71	75
Mal <-> Oxa	46	56	68
Glyoxylate shunt	18	29	40
PEP + CO_2 <-> Oxa	20	32	43
Mal -> Pyr	30	44	56
G6P -> 6PG	19	25	29
6PG -> Ru5P	9	18	27
Ru5P <-> R5P	7	10	14
Ru5P <-> Xu5P	2	8	14
R5P + Xu5P <-> S7P + GAP	2	5	8
GAP+ S7P <-> E4P + F6P	2	5	8
E4P + Xu5P <-> GAP + F6P	0	3	6
ED pathway	0	7	14

Table S2-2. Measured and estimated MID of FAAs_fullset.

Name	Mass isotopomer	Measured MID	Estimated MID
FAA_Ala-57	M	0.217	0.215
	M+1	0.225	0.211
	M+2	0.173	0.173
	M+3	0.385	0.401
FAA_Ala-85	M	0.252	0.254
	M+1	0.270	0.267
	M+2	0.478	0.479
FAA_Asp-57	M	0.072	0.079
	M+1	0.182	0.170
	M+2	0.252	0.257
	M+3	0.284	0.279
	M+4	0.210	0.215
FAA_Asp-85	M	0.108	0.114
	M+1	0.256	0.247
	M+2	0.330	0.330
	M+3	0.306	0.309
FAA_Asp-159	M	0.107	0.114
	M+1	0.251	0.247
	M+2	0.330	0.330
	M+3	0.311	0.309
FAA_Asp302	M	0.254	0.261
	M+1	0.299	0.295
	M+2	0.447	0.444
FAA_Glu-57	M	0.014	0.029
	M+1	0.094	0.093
	M+2	0.207	0.204
	M+3	0.288	0.285
	M+4	0.244	0.240
	M+5	0.152	0.148
FAA_Glu-85	M	0.028	0.043
	M+1	0.140	0.141
	M+2	0.298	0.297
	M+3	0.309	0.303
	M+4	0.225	0.217
FAA_Glu-159	M	0.029	0.043
	M+1	0.141	0.141
	M+2	0.299	0.297
	M+3	0.307	0.303
	M+4	0.223	0.217
FAA_Phe-85	M	0.021	0.021
	M+1	0.047	0.047
	M+2	0.113	0.115
	M+3	0.139	0.134

Table S2-2. *Cont.*

Name	Mass isotopomer	Measured MID	Estimated MID
	M+4	0.170	0.173
	M+5	0.148	0.149
	M+6	0.164	0.166
	M+7	0.123	0.116
	M+8	0.076	0.079
FAA_Phe302	M	0.414	0.418
	M+1	0.138	0.143
	M+2	0.449	0.439
FAA_Gly-57	M	0.425	0.421
	M+1	0.154	0.141
	M+2	0.421	0.439
FAA_Gly-85	M	0.489	0.488
	M+1	0.511	0.512
FAA_Leu-85	M	0.027	0.021
	M+1	0.095	0.087
	M+2	0.189	0.193
	M+3	0.286	0.296
	M+4	0.243	0.247
	M+5	0.159	0.157
FAA_Val-57	M	0.054	0.055
	M+1	0.118	0.111
	M+2	0.206	0.203
	M+3	0.250	0.249
	M+4	0.184	0.190
	M+5	0.187	0.192
FAA_Val-85	M	0.067	0.064
	M+1	0.140	0.135
	M+2	0.319	0.315
	M+3	0.238	0.256
	M+4	0.236	0.230
FAA_Tyr302	M	0.422	0.418
	M+1	0.136	0.143
	M+2	0.442	0.439
FAA_Thr-57	M	0.079	0.079
	M+1	0.179	0.170
	M+2	0.247	0.257
	M+3	0.278	0.279
	M+4	0.216	0.215
FAA_Thr-85	M	0.118	0.114
	M+1	0.253	0.247
	M+2	0.324	0.330
	M+3	0.305	0.309

Supplementary Data 3

Results of metabolic flux analysis using FAAs_Glu+Asp

Table S3-1. Estimated flux distribution and its 95% confidence interval.

**Reaction**	**Lower**	**Best fit**	**Upper**
G6P <-> F6P	55	89	99
F6P -> FBP	67	91	94
FBP <-> DHAP + GAP	67	91	94
DHAP <-> GAP	67	90	93
GAP <-> PGA	157	181	184
PGA <-> PEP	149	172	176
PEP -> Pyr	52	183	233
Pyr -> AcCoA	99	137	187
AcCoA + Oxa -> Cit	6	80	84
Cit ->αKG	6	49	84
αKG -> Suc	0	43	77
Suc <-> Fum	63	74	77
Fum <-> Mal	63	74	77
Mal <-> Oxa	−23	105	154
Glyoxylate shunt	0	31	77
PEP + CO_2 <-> Oxa	−60	−14	115
Mal -> Pyr	0	0	196
G6P -> 6PG	0	10	43
6PG -> Ru5P	0	10	43
Ru5P <-> R5P	4	8	20
Ru5P <-> Xu5P	−4	2	25
R5P + Xu5P <-> S7P + GAP	−1	2	30
GAP + S7P <-> E4P + F6P	−1	2	30
E4P + Xu5P <-> GAP + F6P	−3	0	28
ED pathway	0	0	26

Table S3-2. Measured and estimated MID of FAAs_Glu+Asp+Ala.

Name	Mass isotopomer	Measured MID	Estimated MID
FAA_Asp-57	M	0.072	0.074
	M+1	0.182	0.174
	M+2	0.252	0.253
	M+3	0.284	0.285
	M+4	0.210	0.213
FAA_Asp-85	M	0.108	0.108
	M+1	0.256	0.251
	M+2	0.330	0.333
	M+3	0.306	0.308
FAA_Asp-159	M	0.107	0.108
	M+1	0.251	0.251
	M+2	0.330	0.333
	M+3	0.311	0.308
FAA_Asp302	M	0.254	0.259
	M+1	0.299	0.294
	M+2	0.447	0.447
FAA_Glu-57	M	0.014	0.026
	M+1	0.094	0.091
	M+2	0.207	0.202
	M+3	0.288	0.288
	M+4	0.244	0.246
	M+5	0.152	0.148
FAA_Glu-85	M	0.028	0.039
	M+1	0.140	0.136
	M+2	0.298	0.295
	M+3	0.309	0.310
	M+4	0.225	0.220
FAA_Glu-159	M	0.029	0.039
	M+1	0.141	0.136
	M+2	0.299	0.295
	M+3	0.307	0.310
	M+4	0.223	0.220

Supplementary Data 4

Results of metabolic flux analysis using FAAs_Glu+Asp+Ala

Table S4-1. Estimated flux distribution and its 95% confidence interval.

Reaction	Lower	Best fit	Upper
G6P <-> F6P	59	73	95
F6P -> FBP	72	85	93
FBP <-> DHAP + GAP	72	85	93
DHAP <-> GAP	71	84	92
GAP <-> PGA	162	175	183
PGA <-> PEP	153	167	174
PEP -> Pyr	108	188	222
Pyr -> AcCoA	113	143	179
AcCoA + Oxa -> Cit	70	75	83
Cit ->αKG	7	34	68
αKG -> Suc	0	28	62
Suc <-> Fum	64	69	77
Fum <-> Mal	64	69	77
Mal <-> Oxa	34	110	146
Glyoxylate shunt	10	41	72
PEP + CO2 <-> Oxa	−54	−24	52
Mal -> Pyr	0	0	77
G6P -> 6PG	4	26	40
6PG -> Ru5P	0	25	40
Ru5P <-> R5P	4	13	18
Ru5P <-> Xu5P	−4	12	23
R5P + Xu5P <-> S7P + GAP	−1	7	12
GAP + S7P <-> E4P + F6P	−1	7	12
E4P + Xu5P <-> GAP + F6P	−3	5	10
ED pathway	0	1	20

Table S4-2. Measured and estimated MID of FAAs_Glu+Asp+Ala.

Name	Mass isotopomer	Measured MID	Estimated MID
FAA_Asp-57	M	0.072	0.074
	M+1	0.182	0.174
	M+2	0.252	0.253
	M+3	0.284	0.285
	M+4	0.210	0.213
FAA_Asp-85	M	0.108	0.108
	M+1	0.256	0.251
	M+2	0.330	0.333
	M+3	0.306	0.308
FAA_Asp-159	M	0.107	0.108
	M+1	0.251	0.251
	M+2	0.330	0.333
	M+3	0.311	0.308
FAA_Asp302	M	0.254	0.259
	M+1	0.299	0.294
	M+2	0.447	0.447
FAA_Glu-57	M	0.014	0.026
	M+1	0.094	0.091
	M+2	0.207	0.202
	M+3	0.288	0.288
	M+4	0.244	0.246
	M+5	0.152	0.148
FAA_Glu-85	M	0.028	0.039
	M+1	0.140	0.136
	M+2	0.298	0.295
	M+3	0.309	0.310
	M+4	0.225	0.220
FAA_Glu-159	M	0.029	0.039
	M+1	0.141	0.136
	M+2	0.299	0.295
	M+3	0.307	0.310
	M+4	0.223	0.220

Supplementary Data 5

Results of metabolic flux analysis using FAAs_Glu+Asp+Ala+Phe

Table S5-1. Estimated flux distribution and its 95% confidence interval.

**Reaction**	**Lower**	**Best fit**	**Upper**
G6P <-> F6P	67	75	88
F6P -> FBP	76	84	90
FBP <-> DHAP + GAP	76	84	90
DHAP <-> GAP	75	83	89
GAP <-> PGA	165	174	181
PGA <-> PEP	157	165	172
PEP -> Pyr	123	141	165
Pyr -> AcCoA	125	140	162
AcCoA + Oxa -> Cit	73	77	83
Cit ->αKG	20	40	58
αKG -> Suc	14	34	52
Suc <-> Fum	67	70	77
Fum <-> Mal	67	70	77
Mal <-> Oxa	49	66	88
Glyoxylate shunt	22	36	54
PEP + CO_2 <-> Oxa	0	21	40
Mal -> Pyr	20	41	59
G6P -> 6PG	10	24	32
6PG -> Ru5P		20	32
Ru5P <-> R5P	4	11	15
Ru5P <-> Xu5P	−4	9	17
R5P + Xu5P <-> S7P + GAP	−1	6	10
GAP + S7P <-> E4P + F6P	−1	6	10
E4P + Xu5P <-> GAP + F6P	−3	4	8
ED pathway	0	3	16

Table S5-2. Measured and estimated MID of FAAs_Glu+Asp+Ala+Phe.

Name	Mass isotopomer	Measured MID	Estimated MID
FAA_Ala-57	M	0.217	0.212
	M+1	0.225	0.215
	M+2	0.173	0.171
	M+3	0.385	0.401
FAA_Ala-85	M	0.252	0.244
	M+1	0.270	0.277
	M+2	0.478	0.479
FAA_Asp-57	M	0.072	0.077
	M+1	0.182	0.170
	M+2	0.252	0.259
	M+3	0.284	0.280
	M+4	0.210	0.213
FAA_Asp-85	M	0.108	0.111
	M+1	0.256	0.249
	M+2	0.330	0.331
	M+3	0.306	0.308
FAA_Asp-159	M	0.107	0.111
	M+1	0.251	0.249
	M+2	0.330	0.331
	M+3	0.311	0.308
FAA_Asp302	M	0.254	0.261
	M+1	0.299	0.295
	M+2	0.447	0.444
FAA_Glu-57	M	0.014	0.027
	M+1	0.094	0.092
	M+2	0.207	0.203
	M+3	0.288	0.286
	M+4	0.244	0.244
	M+5	0.152	0.148
FAA_Glu-85	M	0.028	0.040
	M+1	0.140	0.138
	M+2	0.298	0.295
	M+3	0.309	0.308
	M+4	0.225	0.218
FAA_Glu-159	M	0.029	0.040
	M+1	0.141	0.138
	M+2	0.299	0.295
	M+3	0.307	0.308
	M+4	0.223	0.218

Table S5-2. *Cont.*

Name	Mass isotopomer	Measured MID	Estimated MID
FAA_Phe-85	M	0.021	0.019
	M+1	0.047	0.047
	M+2	0.113	0.114
	M+3	0.139	0.136
	M+4	0.170	0.172
	M+5	0.148	0.149
	M+6	0.164	0.165
	M+7	0.123	0.119
	M+8	0.076	0.079
FAA_Phe302	M	0.414	0.412
	M+1	0.138	0.141
	M+2	0.449	0.447
